# Specimen of Reporting

**Published:** 1831-01-01

**Authors:** 


### BARTHOLOMEW'S HOSPITAL.

### TRAUMATIC TETANUS.

*Report.*

"Edward Hogan, labourer, ætat. 29, admitted into St. Luke's Ward under Mr. Vincent, on Saturday, 25th September. The terminal phalanx of the index finger of the right hand was severely lacerated; the integuments di-

### TRAUMATIC TETANUS.

*Analysis.*

Ed. Hogan (aged 29) had the last joint of the fore-finger jammed in a windlass, the bone being fractured, and the soft parts lacerated. He was in health at vided along its outer side and across the articulation; the bone fractured, and its ligamentous connexions extensively torn. The middle finger of the right hand was also slightly injured. He stated, that the injury had occurred that day, and resulted from the fingers having been caught in a windlass, at which he was employed. His health, previous to the accident, was very good, and the several functions were regularly performed.

Some difference of opinion, we understand, arose as to the propriety of at once amputating the injured phalanx. This measure, however, was not proceeded with, and the common poultice was applied. Under this treatment suppuration ensued, and on Tuesday morning, 28th September, the wound discharged abundantly, and looked well. The bowels were at this time rather torpid. During the day he complained of pain from a 'gumboil,' and difficulty of deglutition; but this circumstance escaped particular notice till the evening, when it had become more severe; the internal fauces were relaxed, and rather tender to the touch, and the back of his neck was stiff, uneasy, and slightly swollen. The sister gave him an alum gargle, and rubbed his neck with the common liniment.

29. Rested badly. At seven, A.M. was suddenly seized with a violent tetanic spasm, assuming the forms of trismus and opisthotonos. After the spasm had continued some time, the house-surgeon was called, who directed a drop of croton oil to be repeated every two hours, till the bowels should be relaxed. From that time several spasms occurred, until Mr. Vincent's visit at half past twelve o'clock. The wound was then found dry and pale; the countenance anxious; breathing oppressed and hurried; the neck and abdomen tender to the touch; pulse quick and rather full. Croton oil had not operated. Mr. Vincent ordered the patient a warm bath, two drops of croton oil every second hour till dejections should be procured, and drachm doses of laudanum also, every second hour. The red precipitate to be applied to the wound.

30. Eleven, A.M. Seven drops of croton oil taken, and two common injections administered at alternate periods. Four doses of laudanum used. Bowels scantily moved by the croton oil; injections returned as soon as given. Has slumbered at intervals since the second dose of laudanum. No tetanic spasms through the night, but had one at seven, A.M. and another about ten, A.M. but of less severity than before.

the time of the accident—the injured phalanx was not amputated, but poulticed. In three days, suppuration had taken place, and the discharge was copious and healthy. The bowels were torpid, and the patient complained of difficult deglutition, with stiffness and uneasiness in the back of his neck. *Fourth day.*—Suddenly seized with tetanic spasm, in the forms of trismus and opisthotonos. Croton oil, in one and two drop doses, was repeatedly given, with scanty effects. The wound was dry and pale. Warm bath —croton oil—four drachm doses of laudanum. *Fifth day.*—The tetanic spasms recurred more or less—the pulse was 120—breathing hurried—deglutition extremely difficult—face flushed —cough, and viscid expectoration — anxiously expressive countenance. Croton oil and laudanum to be continued.—Died at four o'clock.

One, P.M. No recurrence of spasm; pulse 120, full; breathing hurried; coughs much, with viscid expectoration. Complains of thirst, but experiences extreme difficulty in deglutition, and the fluids enter the larynx, exciting great distress. The face is flushed, and the conjunctivæ injected; the tongue clean, but dry; skin warm and moist; less tenderness of abdomen; but the neck still feels stiff, and the lower jaw can with difficulty be relaxed. The sterno-mastoid muscles are prominent and rigid; he trembles much, and his countenance is expressive of great anxiety. Croton oil and injections to be repeated as before, and the same doses of laudanum to be resumed if the spasms should recur.

Two, P.M. Another opisthotonic spasm has just taken place, which lasted three minutes, exhibiting the usual phenomena, but with diminished violence; mental functions completely undisturbed.

When we returned, at four, P.M. he had just expired in a spasm, a few minutes after leaving the warm bath."*

*Remarks*. We beg the reader to compare the two columns, and say what useful or necessary word, symptom, or phenomenon we have left out in our analysis? That the case presents no feature of either interest or utility, we positively assert. The whole of the facts lie in a nut-shell. A jammed finger produced tetanus—tetanus was accompanied by constipation—and the result was death, without any dissection. What are we to learn from this?—Nothing! We cannot tell whether amputation would have prevented the tetanus—for tetanus follows amputation—and the wound was kindly suppurating on the third day. There was nothing unusual in the symptoms during life—and, melancholy to relate, there was nothing unusual in its final termination! We hope REPORTERS will endeavour to select cases possessing some interest—and that they will study to convey them to the public in language concise, terse, and expressive. God forbid that we should urge them to imitate those writers who present nothing but the *skeletons* of cases, depriving their *subjects* of all blood, nerve, and muscle—thus wrapping themselves in obscurity, while they are aiming at brevity.

> dum brevis esse laboro
> Obscurus fio.

No. We only wish to divest our *literary subjects, not* of what the printers call "FAT"—for that is positive leanness—but of that VERBAL PLETHORA, that leaden obesity which encumbers the subject, and greatly checks the "MARCH OF INTELLECT."

* Lancet, October 2d, 1830.

